# Characteristics of Soil Microbial Communities and Their Associated Environmental Variables Under Different Vegetation Types in Jianfengling, Hainan

**DOI:** 10.3390/biology15211968

**Published:** 2026-11-02

**Authors:** Xiaoyan Pan, Yiqing Chen, Yuanfeng Gao, Xiaohua Chen, Tingtian Wu, Yuanling Li, Guangyang Li, Ziwei Lin, Haofan Meng, Zongzhu Chen

**Affiliations:** 1 Hainan Academy of Forestry (Hainan Academy of Mangrove), Haikou 571100, China; panxiaoyan@hnaf.ac.cn (X.P.); yiqing.chen@hnaf.ac.cn (Y.C.); gyuanfeng@njfu.edu.cn (Y.G.); xiaohua.chen@hnaf.ac.cn (X.C.); tingtian.wu@hnaf.ac.cn (T.W.); yuanling.li@hnaf.ac.cn (Y.L.); li15130574839@163.com (G.L.); linziwei2019@email.szu.edu.cn (Z.L.); 2 Key Laboratory of Tropical Forestry Resources Monitoring and Application of Hainan Province, Haikou 571100, China; 3 School of Tropical Agriculture and Forestry, Hainan University, Haikou 571100, China; m15949826300@163.com

**Keywords:** vegetation type, soil microorganism, microbial community, diversity, high-throughput sequencing

## Abstract

Soil microorganisms play essential roles in maintaining the function and stability of forest ecosystems. In this study, conducted in the Jianfengling Nature Reserve in Hainan, we performed Illumina high-throughput sequencing, in combination with principal coordinate analysis (PCoA), redundancy analysis, and other statistical methods, to compare the composition, community structure, and diversity of the soil microbiota in four types of forest vegetation, namely, monsoon forest, lowland rainforest, montane rainforest, and artificial forest (AF), and to identify the environmental variables associated with differences among the forest types. Different vegetation types differed significantly with respect to pH, total phosphorus content (TP), elevation, and concentrations of manganese (Mn) and iron (Fe). In contrast, no significant differences were observed in soil water content, bulk density, total nitrogen (TN), and total organic carbon (TOC) content. Compared to AF, natural forests were characterized by higher concentrations of TN, TOC, TP, Mn, and Fe, although the differences in TN and TOC were not statistically significant. For all four of the assessed vegetation types, Pseudomonadota, Actinomycetota, Acidobacteriota, and Verrucomicrobiota were identified as the dominant bacterial phyla, whereas Ascomycota, Basidiomycota, and Mucoromycota were predominant among the fungal phyla, indicating that these phyla are capable of surviving in different soil ecosystems. Although no significant differences in bacterial diversity were detected among vegetation types, PCoA revealed pronounced differentiation in bacterial community composition (PERMANOVA, *p* = 0.0001). Notably, within-group multivariate dispersion also differed significantly (PERMDISP, *p* = 0.0129), indicating that the observed compositional differences may be associated with the combined effects of centroid location effect and within-group multivariate dispersion. In contrast, significant differences were observed in fungal diversity, whereas differences in fungal community composition were not significant in the PCoA (PERMANOVA, *p* = 0.1777). These results suggest that vegetation type primarily influenced fungal diversity rather than fungal community structure. Mn concentration exhibited the highest marginal explanatory power for the variation in the bacterial community, with a significant effect; it was positively correlated with the ACE, Chao1, and Simpson indices, and negatively correlated with the Shannon index. TOC exhibited the highest marginal explanatory power within the fungal community, showing a negative correlation with the ACE, Chao1, and Shannon indices and a positive correlation with the Simpson index. Collectively, these findings provide insights into microbial differentiation within forest soils associated with different vegetation types and highlight distinct patterns in bacterial and fungal diversity, which will potentially serve as a basis for the development of conservation strategies.

## Simple Summary

This study analyzed the differences in microbial communities associated with vegetation types using high-throughput sequencing and identified environmental variables related to community variation. Four vegetation types in the Jianfengling Nature Reserve in Hainan were evaluated, namely monsoon forests, lowland rainforests, montane rainforests, and artificial forests. Natural forests (monsoon forests, lowland rainforests, and montane rainforests) had higher soil nutrient contents than artificial forests. The dominant bacterial phyla across all four vegetation types were Pseudomonadota, Actinomycetota, Acidobacteriota, and Verrucomicrobiota, while the dominant fungal phyla were Ascomycota, Basidiomycota, and Mucoromycota. For bacteria, the ACE, Chao1, and Simpson indices were higher in natural forests than in artificial forests, whereas the Shannon index was lower in natural forests than in artificial forests. The fungal diversity indices (ACE, Chao1, Shannon) peaked in the lowland rainforest, while the Simpson index dipped lowest there. Significant differentiation in bacterial communities was observed under different vegetation types, whereas fungal communities displayed relatively concentrated patterns. Manganese concentration was a significant environmental variable associated with the variation in bacterial communities, while total organic carbon was the environmental variable most strongly associated with the variation in fungal communities. These findings provide baseline information on changes in soil microbial communities under different vegetation types in Jianfengling Nature Reserve, offering potentially useful information for future monitoring and restoration research.

## 1. Introduction

Tropical rainforests are the most biodiverse, structurally complex, and functionally significant terrestrial ecosystems in the world, playing vital roles in global carbon balance, hydrological regulation, and biogeochemical cycles [1,2]. Soil is the core medium that sustains the ecological functions of tropical rainforest ecosystems. Diverse soil microbial communities regulate organic matter decomposition, humus formation, and biogeochemical cycles of essential elements, such as carbon, nitrogen, and phosphorus, serving as a key driver of material cycling and energy flow within the ecosystem [3,4,5]. In forest ecosystems, aboveground vegetation and soil microorganisms exhibit complex interactions. Changes in vegetation directly alter the quantity and quality of litter input and root exudates, thereby influencing nutrient availability and substrate composition, which ultimately leads to shifts in microbial community structure and function. In short, vegetation changes are the primary factors influencing the soil microbial composition, community structure, and function [6,7,8,9,10]. Studies have shown that soil properties vary across different vegetation types, which provide diverse habitats for microorganisms, regulating the structure and diversity of microbial communities and ultimately influencing the spatial distribution of microorganisms [11,12,13]. For example, Xi et al. [10] found that broadleaf forests have a higher Shannon index than those of coniferous forests and mixed coniferous–broadleaf forests. Li et al. [14] pointed out that fungal species richness is lower in artificial forests than in natural forests.

The Hainan Tropical Rainforest is China’s most diverse, best-preserved, and extensive contiguous area of continental island tropical rainforest, regarded as a rich source of tropical biodiversity and genetic resources [15]. The Jianfengling Tropical Rainforest is a vital constituent of the Hainan Tropical Rainforest. Under the impacts of climate change and human activities, the Jianfengling region has experienced significant warming alongside severe biodiversity loss, threatening the security and functional stability of the ecosystem [16,17]. Given the complex relationship between vegetation and soil microorganisms, elucidating the community structure of soil microorganisms and their associated environmental variables across different vegetation types is crucial for accurately assessing the health of the Jianfengling ecosystem and predicting its response to climate change. The Jianfengling region features a rich variety of vegetation types, a complex and diverse vegetation gradient, and distinct vertical zonation, providing an ideal “natural laboratory” for studying soil microbial ecological processes under different vegetation types. Several studies have focused on this region. For example, Chen et al. [18] investigated soil carbon-fixing microorganisms. Huang et al. [19] and Lin et al. [20] independently compared soil bacteria and fungi at different depths in natural forests along an elevation gradient. However, no studies have evaluated soil microbial systems across different vegetation types in the Jianfengling area, and few studies have involved artificial forests. Against this background, setting the Jianfengling section of the Hainan Tropical Rainforest National Park as the study area, soil samples were collected across four vegetation types: monsoon forests (MF), lowland rainforests (LR), montane rainforests (MR), and artificial forests (AF). Using high-throughput sequencing, this study aimed to elucidate the soil microbial composition, community structure, and diversity characteristics across different vegetation types in the Jianfengling region and to determine the relative contributions of environmental variables to the community composition and variation. These findings provide foundational data for subsequent monitoring and ecological restoration.

## 2. Study Area and Methods

### 2.1. Overview of the Study Area

The Jianfengling section of Hainan Tropical Rainforest National Park is located in the southwestern part of Hainan Island (108°44′–109°4′ E, 18°34′–18°52′ N). It is situated in the tropical rainforest climate zone, covering an area of 678.43 km^2^ at an elevation of 1412 m. The average annual rainfall of 2651.6 mm, and the average annual temperature is 24.5 °C [18,20,21]. It has 2286 species of wild vascular plants, with a forest coverage as high as 98% (https://www.hntrnp.com/, accessed on 18 August 2026). The soil types are predominantly Acrisols and Alisols (http://soil.geodata.cn, accessed on 27 October 2026).

### 2.2. Sample Collection

**Figure 1:**
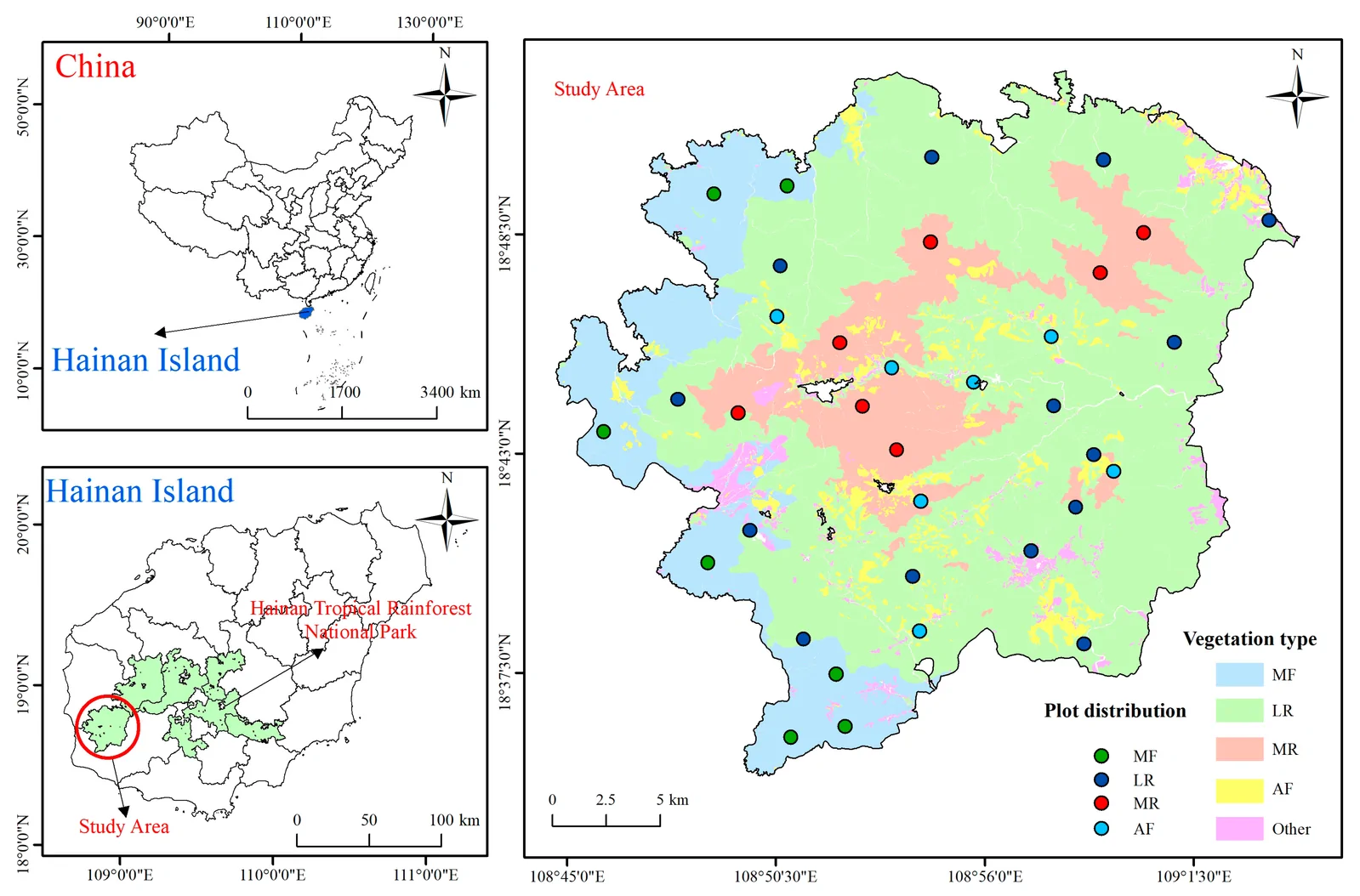
Distribution of sampling points. MF, LR, MR, and AF represent monsoon forests, lowland rainforests, montane rainforests, and artificial forests, respectively.

Based on the area distributions of the vegetation types of interest in the Jianfengling section, 14, 7, 7, and 7 replicate plots (20 m × 20 m) were established for the LR, MF, MR, and AF, respectively, in November 2024. This process yielded 35 sampling plots. Among them, MF spans an elevation range of 150–460 m, LR spans 190–800 m, AF spans 315–815 m, and MR spans 875–1130 m. The elevation ranges of MF, LR, and AF overlap with one another. Sample plots of the same vegetation type must be separated by a minimum distance of 2.5 km to represent distinct forest communities. Five-point sampling was performed by collecting soil at a depth of 0–20 cm from the four corners and center of each plot to obtain a composite soil sample [10,18,19,20]. The fresh soil was cleaned of plant roots, stones, and other impurities and then divided into two subsamples [8,18,19,20]. One subsample was placed in a car refrigerator and transported back to the laboratory for storage at −80 °C for the Illumina high-throughput sequencing analysis and the other one was left to air-dry under ambient conditions and subsequently used for determining soil physicochemical properties. All natural forest sample plots were likewise established within broad-leaved forests. All AF sample plots were dominated by *Pinus caribaea* Morelet. They were planted in the 1990s and maintained under artificial management until the early 2000s, after which they were withdrawn from management and allowed to grow naturally. The distribution of sampling points is shown in Figure 1.

### 2.3. Methods of Analysis

#### 2.3.1. Determination of Soil Physicochemical Properties

The soil water content (SWC) was determined using the oven-drying method, in which fresh soil was oven-dried at 105 °C for 24 h [22]. Bulk density (BD) and pH were determined according to the methods specified in the national standards NY/T 1121.4-2006 and NY/T 1121.2-2006, respectively. Total organic carbon (TOC), total phosphorus (TP), and total nitrogen (TN) were determined according to the methods specified in NY/T 1121.6–2006, LY/T 1232-2015, and LY/T 1228-2015, respectively. The concentrations of iron (Fe) and manganese (Mn) were measured according to the methods specified in LY/T 3129-2019.

#### 2.3.2. Soil DNA Extraction and Metagenomic Sequencing and Analysis

Total DNA was extracted from fresh soil using the PF Mag-Bind Soil DNA Kit (Omega Bio-Tek, Norcross, GA, USA) following the manufacturer’s instructions. DNA purity and concentration were measured using a NanoDrop 2000 spectrophotometer (Thermo Fisher Scientific, Waltham, MA, USA) and TBS-380 fluorometer (Turner Biosystems, Sunnyvale, CA, USA), respectively, and DNA integrity was verified via 1% agarose gel electrophoresis. Subsequently, a DNA library containing insert fragments of approximately 400 bp was constructed using a Covaris M220 Focused-ultrasonicator (Covaris, Woburn, MA, USA). Metagenomic sequencing was conducted using the Illumina NovaSeq X Plus System at Majorbio Biomedical Technology Co., Ltd. (Shanghai, China).

Raw sequencing reads were quality-filtered using Fastp (version 0.23.0) [23]. Bases with Phred quality scores ≥ 20 were considered qualified. Reads shorter than 50 bp after preprocessing or those containing more than five ambiguous bases were discarded. Quality trimming was performed using a 50 bp sliding window scanned from the 5′ to the 3′ end. Upon encountering a window with a mean Phred quality score < 20, the window and all downstream bases were removed. MEGAHIT (version 1.2.9) was used to assemble clean reads [24], and contigs shorter than 500 bp were removed [10]. Subsequently, Prodigal (version 2.6.3) was used to predict open reading frames (ORFs) within the contigs and translate these into amino acid sequences [25]. CD-HIT (version 4.6.1) was used to construct a non-redundant gene set based on a sequence similarity of 90% and coverage of 90% [26], and with reference to the NR database, Diamond (version 2.0.13) was used to align the amino acid sequences of the non-redundant gene set to obtain the corresponding species annotations. For each species, relative abundance was calculated as the total abundance of genes annotated to that species. Transcripts Per Million normalization was applied for the abundance calculation. Sample information in detail is shown in Table S1.

#### 2.3.3. Data Processing

Excel 2010 was used to organize and analyze the data. One-way analysis of variance (ANOVA) was performed using SPSS Statistics 19.0 on environmental variables and microbial diversity among different vegetation types, provided that the data met the assumptions of residual normality and homogeneity. Duncan’s multiple range test (*p* < 0.05) was subsequently applied to determine significant differences among vegetation types. When the data did not satisfy the normality assumption, the non-parametric Kruskal–Wallis H test was used instead, followed by Dunn’s post hoc test with multiple comparisons for pairwise differences. A principal coordinate analysis (PCoA) based on Bray–Curtis distances was performed to examine differences in the microbial community structure across vegetation types. Subsequently, permutational multivariate analysis of variance (PERMANOVA) and permutational multivariate analysis of dispersion (PERMDISP) were conducted to test the statistical significance of these differences. By combining Spearman’s rank correlation coefficient and the Benjamini–Hochberg false discovery rate (BH-FDR), we explored the relationships between environmental variables and microbial communities. The Variance Inflation Factor (VIF) test was used to exclude environmental variables with severe multicollinearity (VIF > 10). Redundancy analysis (RDA) was employed to examine the association between environmental variables and the variation in microbial community composition. Based on the constructed RDA model, a marginal permutation test was performed to evaluate the marginal explanatory contribution (marginal R^2^) of each environmental variable. Statistical significance of each variable was determined via a Monte Carlo permutation test, and the significance values were corrected using the BH-FDR procedure. Data analyses and visualizations were performed using Python (version 3.10). The diversity index was calculated as described by Cao et al. [27], in which the formula for the Simpson index is given by (1). A higher Simpson index indicates lower community diversity.

(1) $$D_{simpson}=\frac{\sum_{i=1}^{S_{obs}}n_{i}\left(n_{i}-1\right)}{N\;(N-1)}$$

where *S_obs_* is the number of species observed, *n_i_* is the number of sequences contained in the *i*th species, and *N* is the total number of sequences.

## 3. Results

### 3.1. Environmental Variables of Different Vegetation Types

A comparison of associated environmental variables across different vegetation types revealed that pH, TP, Mn and Fe concentrations, and El differed significantly among vegetation types (Table 1). Specifically, pH was the highest in MF and lowest in LR; TP in MR and MF was significantly higher than that in LR and AF; and both Mn and Fe followed the order MR > MF > LR > AF, whereas El followed the order MR > AF > LR > MF. In contrast, SWC, BD, TN, and TOC showed no significant differences among the four vegetation types, although MR exhibited the lowest SWC with the highest BD, and both TN and TOC followed the order MR > MF > LR > AF.

### 3.2. Differences in Soil Microbial Community Composition at the Phylum Level Associated with Vegetation Type

**Figure 2:**
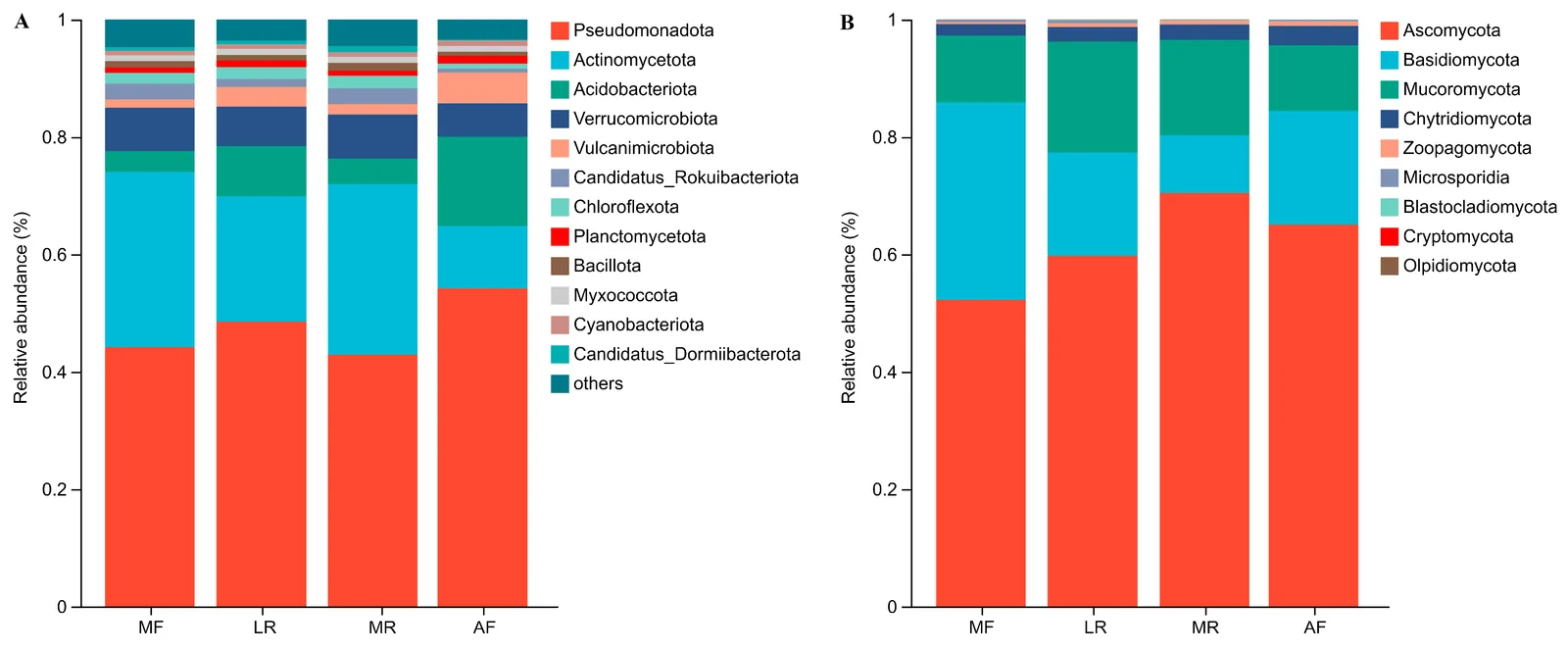
Relative abundances of soil (**A**) bacterial and (**B**) fungal phyla across different vegetation types. MF, LR, MR, and AF represent monsoon forests, lowland rainforests, montane rainforests, and artificial forests, respectively.

At the phylum level, the dominant bacteria across all four vegetation types were Pseudomonadota (42.89–54.20%), Actinomycetota (10.61–29.86%), Acidobacteriota (3.58–15.23%), and Verrucomicrobiota (5.65–7.50%), with a total relative abundance exceeding 80% across all vegetation types (Figure 2A, Table S2). The abundance of Pseudomonadota followed the order: AF > LR > MF > MR. AF exhibited lower abundances of Actinomycetota and Verrucomicrobiota than those of other vegetation types but a higher abundance of Pseudomonadota and Acidobacteriota. Ascomycota (52.25–70.41%), Basidiomycota (9.86–33.66%), and Mucoromycota (11.18–18.85%) dominated the fungal community, with a total relative abundance exceeding 90% across all vegetation types (Figure 2B, Table S3). Compared with the other vegetation types, MF showed a lower abundance of Ascomycota but a higher abundance of Basidiomycota.

### 3.3. Differences in Microbial Diversity at the Genus Level Associated with Vegetation Type

As shown in Table 2, vegetation type, elevation and soil physicochemical properties as well as their interactions showed stronger associations with fungal diversity than with bacterial diversity. For bacteria, the four diversity indices did not differ significantly across different vegetation types. Specifically, the richness estimators ACE and Chao1 were higher in natural forests than in AF, and the Simpson index (where higher values denote lower diversity) was also higher in natural forests, whereas the Shannon index, which integrates both richness and evenness, was higher in AF. For fungi, the four diversity indices differed significantly between the four vegetation types. ACE and Chao 1 showed the order of LR > AF > MR > MF, whereas the Shannon index was highest in LR and lowest in MF. The pattern of variation in the Simpson index was the opposite of that of the Shannon index.

### 3.4. Microbial Community Structure Differences Associated with Vegetation Type at the Phylum Level

**Figure 3:**
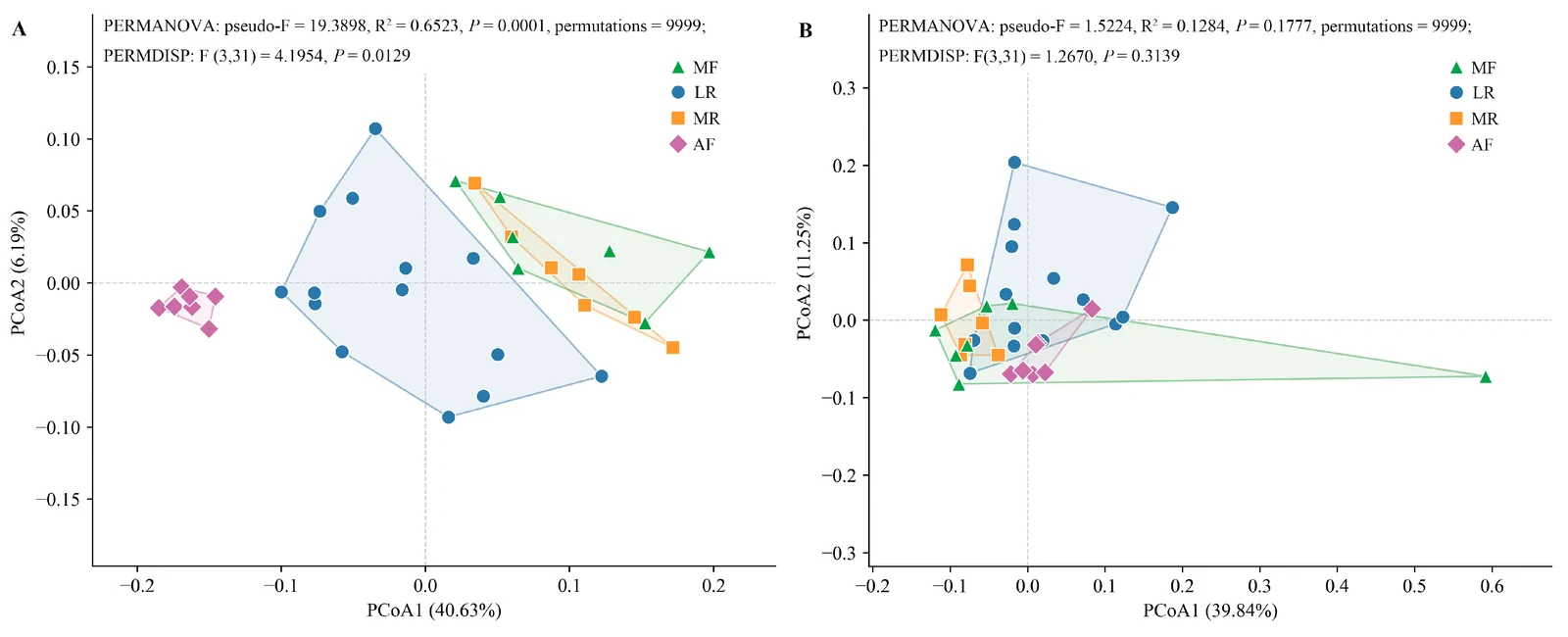
PCoA plots of soil (**A**) bacterial and (**B**) fungal communities under different vegetation types. MF, LR, MR, and AF represent monsoon forests, lowland rainforests, montane rainforests, and artificial forests, respectively.

In the PCoA, the first principal component explained 40.63% of the variation in the bacterial community, and the second principal component explained 6.19%. Together, they account for 46.82% of the total community variation (Figure 3A). LR and AF were clearly separated from the other vegetation types, whereas MR and MF showed a substantial overlap. However, the overall community structure differed significantly (PERMANOVA, *p* = 0.0001), but the within-group multivariate dispersion also differed significantly (PERMDISP, *p* = 0.0129). For the fungal community, the first and second principal components explained 39.84% and 11.25% of the variance, respectively, together accounting for 51.09% of the total variation (Figure 3B). However, compared with those of the bacterial community, data points for the fungal community were more concentrated in the PCoA plot, and the differences in the soil fungal community structure among the different vegetation types were not significant (PERMANOVA, *p* = 0.1777). Additionally, the within-group multivariate dispersion also showed no significant difference (PERMDISP, *p* = 0.3139).

### 3.5. Correlation Analysis of Soil Microbial Communities with Environmental Variables at the Phylum Level

**Figure 4:**
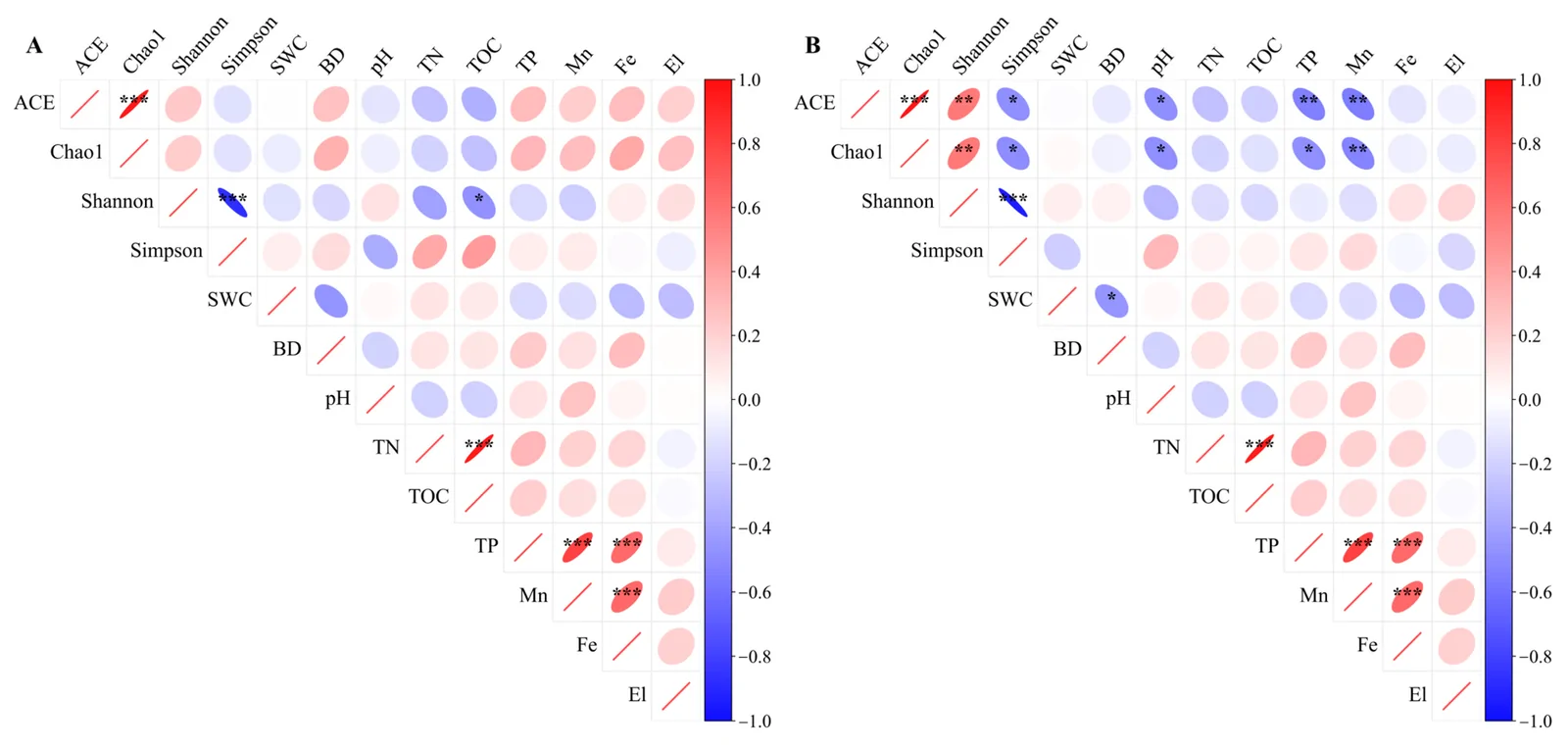
Correlations between diversity indices of soil (**A**) bacteria and (**B**) fungi and environmental variables. * BH-q < 0.05, ** BH-q < 0.01, and *** BH-q < 0.001. SWC, BD, TOC, TN, and TP denote soil water content, bulk density, total organic carbon, total nitrogen, and total phosphorus, respectively. Mn and Fe represent manganese and iron concentrations, respectively. El indicates elevation.

As shown in Figure 4, the ACE and Chao1 indices for bacteria were positively correlated with BD, TP, and El as well as with Mn and Fe concentrations, whereas the two indices were negatively correlated with SWC, pH, TN, and TOC. The Shannon index was positively correlated with pH, Fe concentration and El but negatively correlated with SWC, BD, TN, TOC, TP and Mn concentration, whereas the Simpson index generally showed the opposite trend. For fungi, the ACE and Chao1 indices were negatively correlated with all environmental variables except SWC. The Shannon index was positively correlated with SWC, BD, Fe concentration, and El but negatively correlated with pH, TN, TOC, TP, and Mn concentration, whereas the Simpson index generally exhibited the opposite pattern.

**Figure 5:**
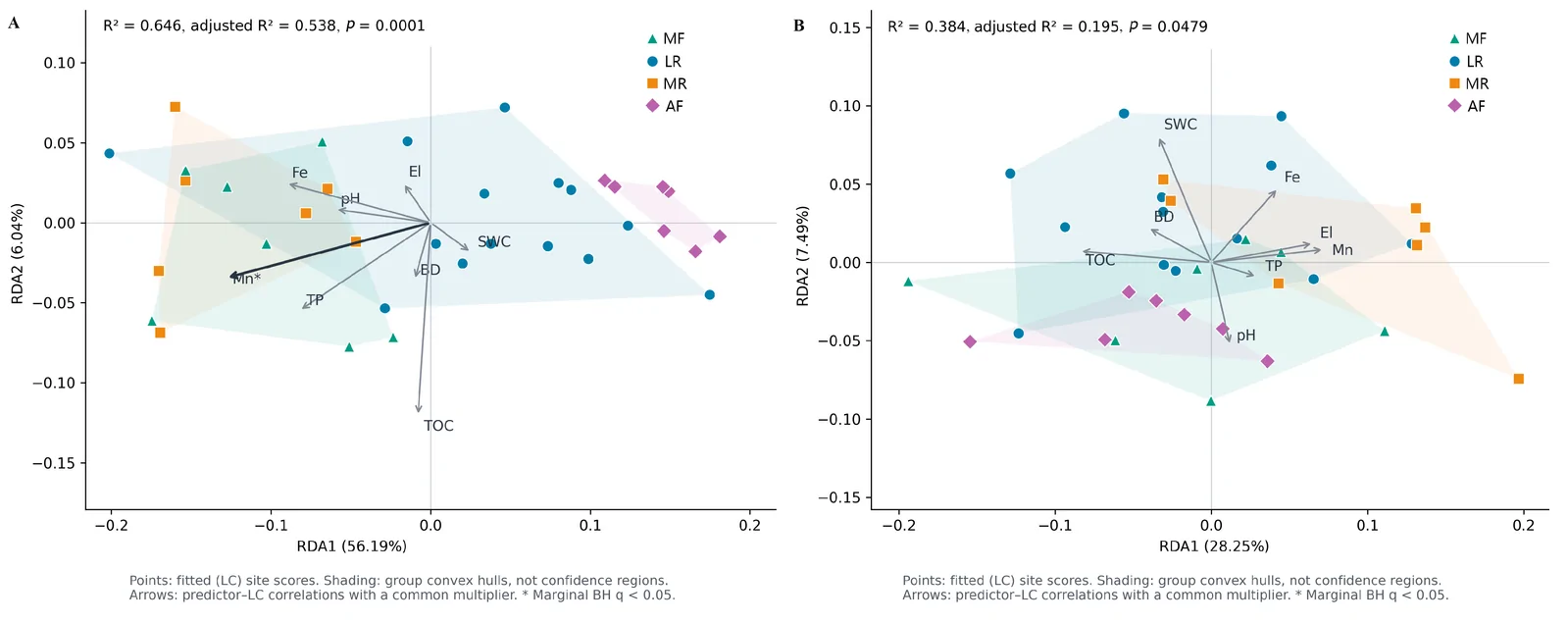
RDA of the relationship between soil environmental variables and (**A**) bacterial and (**B**) fungal communities under different vegetation types. MF, LR, MR, and AF represent monsoon forests, lowland rainforests, montane rainforests, and artificial forests, respectively. SWC, BD, TOC, TN, and TP denote soil water content, bulk density, total organic carbon, total nitrogen, and total phosphorus, respectively. Mn and Fe represent manganese and iron concentrations, respectively. El indicates elevation.

As the VIF values of TN exceeded 10, they were removed prior to RDA. The RDA results revealed that the ordination axis explanatory rates and primary associated environmental variables differed between the bacterial and fungal communities (Figure 5). For the bacterial community, the eigenvalues of the first and second axes were 56.19% and 6.04%, respectively, with a cumulative explanation for 62.23% of the variation in soil bacterial communities. Most environmental variables were concentrated on the left side of the first canonical axis (RDA1), where the MF and MR sampling sites were clustered. TOC, TP, Mn, and Fe concentrations were closely associated with the bacterial community. RDA1 explained 28.25% of the fungal community; its left side was primarily influenced by TOC, SWC, and BD, where the LR and AF sampling sites were clustered, whereas its right side was primarily influenced by pH, Fe and Mn concentrations, and El and TP, where the MR sampling site clustered. TOC and SWC are closely associated with the fungal community. RDA2 explained only 7.49% of community variation, representing a secondary gradient with little discriminatory power.

Based on the marginal effects (Table 3), the environmental variables associated with the bacterial community were ranked in descending order of their marginal explanatory contributions as follows: Mn concentration, Fe concentration, El, TOC, pH, TP, SWC, and BD, among which Mn concentration exerted a significant effect on the bacterial community (BH-q = 0.0024). For the fungal community, the environmental variables, ranked in descending order, were TOC, SWC, Mn concentration, BD, Fe concentration, El, pH, and TP. Although none of the individual environmental variables retained statistical significance after BH-FDR correction (all BH-q > 0.05), TOC exhibited the highest marginal explanatory power among all measured variables. Nevertheless, the overall RDA model was statistically significant (*p* = 0.0479), indicating that the combined effects of environmental variables are still significantly associated with the fungal community. It should be noted that these rankings reflect marginal contributions rather than independent ecological effects, and the environmental variables may be correlated with one another.

## 4. Discussion

### 4.1. Soil Nutrient Differences Associated with Vegetation Type

Plant communities can improve soil fertility, and soil nutrient concentrations differ significantly among vegetation types [28,29]. In this study, the concentrations of five soil nutrients (TN, TOC, TP, Mn, and Fe) were higher in natural forests than in AF, indicating that soil fertility in natural forests was higher than that in AF, which is consistent with the results of previous studies [30,31]. The natural forests in this study had a complex species composition, a relatively dense canopy that intercepted rainfall effectively, well-developed root systems, and a thick litter layer. Canopy interception is positively correlated with stand closure, and natural forests generally have higher closure than that of artificial forests. In addition, root networks of the former are more developed than those of artificial forests, promoting nutrient uptake by plants [32,33,34,35]. These findings provide partial support for the present results. Unfortunately, this study only compared soil nutrient concentrations in the surface layer and could not provide direct process-based evidence to quantitatively assess the nutrient retention capacities of the two stand types.

### 4.2. Differences in Microbial Diversity Associated with Elevation

Elevation affects water and temperature conditions at the regional scale and is a key factor influencing microbial composition and community structure [36,37]. Soil microbial diversity captures the abundance, richness, and evenness of soil microorganisms. Numerous studies based on high-throughput sequencing have demonstrated that the diversity of soil fungal and bacterial communities decreases with increasing elevation [19,38,39,40,41,42]. In this study, the elevations of the four vegetation types generally followed the order MR > AF > LR > MF. However, microbial diversity did not exhibit a corresponding increase along this gradient. In particular, bacterial diversity did not show significant variation along the elevation gradient, consistent with the findings of Fierer et al. [43] and Yu et al. [44]. Fungal diversity was lowest in MF, which had the lowest elevation, while the other vegetation types generally showed a decrease in diversity with increasing elevation, which is not entirely consistent with previous findings. This discrepancy may be associated with pH, which exhibited a pattern opposite to that of diversity, with significant differences observed across the different vegetation types. Furthermore, the Jianfengling region in Hainan has a warm climate year-round, with hot and rainy summers. Heat and rainfall tend to accumulate in low-elevation areas. Excessively high temperatures and humidity can limit the growth of certain microorganisms, and intensive human disturbance in low-elevation areas reduces plant diversity, indirectly leading to decreased microbial diversity.

### 4.3. Differences in Soil Microbial Diversity Associated with Soil Environmental Variables

The ACE and Chao1 indices are estimators of community richness; the higher their values, the higher the species richness of the community [45]. The Shannon index reflects both species richness and evenness, with higher values indicating greater community diversity [45]. The Simpson index is sensitive to evenness; in this study, lower Simpson values indicate higher community diversity. For bacteria, the ACE, Chao1, and Simpson indices were higher in natural forests than in AF, whereas the Shannon index was higher in AF than in natural forests. It is speculated that this pattern may reflect the complexity of the community structure. Complex natural forests can provide a diverse range of ecological niches and thus accommodate a greater number of species, some of which are, however, clearly dominant. Comparatively, the community structure in artificial forests is relatively simple, with species being more evenly distributed. Sensitivity to soil nutrients may also be associated with differences in diversity. In this study, after BH-FDR correction, the Mn concentration emerged as the sole soil variable with a significant marginal effect, showing the strongest association with bacterial community composition and structure. Manganese is a key trace element in soil that participates in various redox processes and enzymatic reactions within microorganisms, serves as a cofactor for multiple enzymes, and indirectly shapes microbial communities by influencing microbial metabolic processes [46,47]. The results revealed that the Mn concentration in AF was significantly lower than that in natural forests. They exhibited positive correlations with the ACE, Chao1, and Simpson indices, while showing a negative correlation with the Shannon index. These findings suggest that within a certain range, higher Mn concentrations facilitate increased species richness of bacterial communities yet may promote the growth of dominant species and reduce species evenness. This could be attributed to the fact that, as a micronutrient, Mn at appropriate concentrations can promote the growth and proliferation of diverse bacterial taxa, thereby increasing the number of species, whereas the distribution of Mn may be tightly coupled with specific soil microenvironments, creating ecological niches favorable to particular dominant taxa. Iron is one of the most abundant trace elements in soil and serves as a cofactor for various enzymes. Fe respiration is an important metabolic pathway for many bacteria [48], which is positively correlated with the ACE, Chao1, and Shannon indices and negatively correlated with the Simpson index, indicating that an increase in Fe concentration is conducive to the development of bacterial community diversity. For fungi, TOC showed the highest marginal explanatory power for community composition in the RDA, although its effect did not remain statistically significant after BH-FDR correction. Correlation analysis showed that TOC was negatively correlated with the ACE, Chao1, and Shannon indices, and positively correlated with the Simpson index. This may be attributed to excessive litter input, which led to the overaccumulation of TOC. Rapidly proliferating eutrophic-dominant microorganisms occupy a large number of ecological niches, squeezing the living space of rare species, thereby resulting in a decline in diversity. Unfortunately, this study could not provide direct evidence to verify this speculation; however, it is highly consistent with the findings of Ran et al. [49]. Furthermore, although TOC showed no significant difference among different vegetation types, it remained the most strongly associated environmental variable with the fungal community. This indicates that within the same vegetation type, obvious community shifts occurred along the gradient of TOC from high to low. Apart from TOC, SWC accounted for the largest marginal explanatory power for fungal community composition; however, its correlation was very low in the Spearman analysis, suggesting that SWC may regulate the composition and distribution of fungal communities in synergy with other variables. Mn was correlated with fungal communities similar to TOC, but was more significant, indicating that increasing Mn concentration inhibits fungal diversity, which is consistent with previous studies [50,51,52,53]. In contrast to bacteria, pH and TP showed significant negative correlations with the fungal ACE and Chao1 indices, although their marginal explanatory power was very low. This may be attributed to two reasons: multicollinearity among environmental variables in the fungal community, and a large number of undetected variables that have significant marginal effects on microbial community but relatively low marginal effects on conventional indicators. It should be emphasized that soil environments are closely related to microbial communities. These relationships are consistent with evidence that changes in soil conditions associated with plant and management interventions can modify microbiological attributes, reinforcing the need to interpret microbial community variation within the broader soil–plant environmental context rather than as an isolated response to vegetation identity [54].

### 4.4. Differences in Microbial Community Composition and Structure Associated with Vegetation Type

Diversity indices reflect species richness and evenness within a community, whereas PCoA evaluates the degree of similarity or dissimilarity in species composition among communities; the two measure different dimensions of community diversity. Although we detected a substantial variation in fungal diversity among the different vegetation types, the data points were relatively concentrated in the PCoA plot, thus indicating that although fungal species richness and evenness differed markedly according to the type of forest vegetation, the overall structure and composition of the fungal community did not show any substantial differentiation (PERMANOVA, *p* = 0.164). These findings would thus tend to indicate that whereas vegetation type may have a considerable influence on fungal community diversity, it does not play a prominent role in community composition. In contrast, although we failed to detect significant differences in bacterial diversity among the assessed vegetation types, PCoA revealed a significant differentiation in community composition (PERMANOVA, *p* = 0.0001). Notably, the within-group multivariate dispersion of bacteria also differed significantly (PERMDISP, *p* = 0.0129), suggesting that the observed differences in bacterial community composition may be confounded by the combined effects of the centroid location effect associated with vegetation type and the within-group dispersion effect. The association between plant cover and belowground microbial organization is also supported by evidence from contrasting plant-management systems, in which changes in plant composition and rotation can restructure soil microbial communities, emphasizing the close coupling between aboveground vegetation and belowground microbial assemblages [55]. At the phylum level, Pseudomonadota, Actinomycetota, Acidobacteriota, and Verrucomicrobiota were identified as dominant bacterial taxa in the assessed forest soils, whereas Ascomycota, Basidiomycota, and Mucoromycota predominated among the fungal taxa, which is consistent with the findings of Huang et al. [19] and Lin et al. [20] in the Jianfengling area. Notably, the contents of soil TN and TP reported in the two aforementioned studies were substantially higher than those recorded in the present study, whereas pH and SWC were substantially lower, thereby providing evidence that organisms within these phyla have the capacity to adapt to environmental change. In contrast, although the dominant phyla identified in this study are similar to those reported by Huang et al. and Lin et al., their relative abundances differed considerably in different soil environments, thus indicating that the composition of the soil microbial community and its patterns of distribution are a consequence of environmental filtering.

### 4.5. Bidirectional Feedback Mechanisms of the Vegetation–Soil–Microbial Community

Existing studies have demonstrated that vegetation, soil, and microbial communities constitute an integrated whole characterized by mutual coupling and bidirectional feedback within ecosystems [56]. Through litterfall and root exudates, plants continuously input energy substrates into the soil, thereby modifying the soil environment and regulating microbial communities [57,58,59]. Conversely, soil microorganisms act as decomposers and mineralize litterfall and organic matter to release nutrients available for plant uptake, which in turn regulates soil nutrient availability and plant growth [56,60]. Based on this bidirectional regulatory mechanism, aboveground vegetation communities and belowground microbial communities can mutually shape and co-evolve.

Within the context of this study, the aforementioned bidirectional mechanism has direct ecological significance. The natural forests and plantations investigated in this study differ in terms of tree species composition, canopy structure, root development, and litter thickness. These differences may contribute to variations in the quality and quantity of litterfall entering the soil as well as in the concentration and composition of root exudates, which in turn affect the soil nutrient content in natural forests and plantations. Through differential soil environmental filtering, the composition and distribution patterns of microbial communities were found to differ. In contrast, soil microorganisms may feedback on vegetation growth and regeneration by influencing nutrient mineralization, organic matter turnover, and niche supply, thereby forming a closed feedback loop. Consequently, the variations in soil environmental variables, microbial composition, and diversity observed in this study do not always align consistently with vegetation type changes, because the soil microenvironments under different vegetation types may result from long-term interactions between vegetation and microorganisms, rather than being determined unidirectionally by vegetation type alone. Admittedly, this study was based on static observations of surface soil nutrients and cannot yet provide process-level direct evidence to quantify the direction and intensity of this feedback.

### 4.6. Limitations of the Present Study

The limitations of the present study should be acknowledged from four perspectives. First, the current work primarily investigated the species composition and diversity of the microbial community, and examined its relationship with soil physicochemical properties; however, functional gene analysis was not conducted, which warrants more in-depth investigation in future research. Second, process-level direct evidence was not obtained to quantitatively verify the speculations in Section 4.1 and Section 4.3 and the bidirectional feedback mechanism proposed in Section 4.5. Third, because complete information on soil texture and parent material for the studied plots was not collected, the potential confounding effects of edaphic variables on the observed relationships cannot be entirely excluded. Finally, given that only a single sampling campaign was carried out, the findings may not fully capture the year-round dynamics of the microbial community.

## 5. Conclusions

(1) Significant differences were observed in pH, TP and El as well as concentrations of Mn and Fe among vegetation types, while no noticeable differences were found for SWC, BD, TN, and TOC. The concentrations of five soil nutrients (TN, TOC, TP, Mn, and Fe) were higher in natural forests than in AF, although the differences in TN and TOC were not statistically significant. The MR exhibited the highest El, along with the highest BD, TOC, TN, TP, Mn, and Fe, while its SWC was the lowest. The pH was highest in MF and lowest in LR.
(2) The dominant bacterial and fungal phyla were the same across different vegetation types; however, their abundances differed markedly. The dominant bacterial phyla included Pseudomonadota, Actinomycetota, Acidobacteriota, and Verrucomicrobiota, while the dominant fungal phyla were Ascomycota, Basidiomycota, and Mucoromycota. These findings demonstrate that these phyla are capable of surviving across diverse soil ecosystems and that vegetation types can regulate niche spaces by altering the soil environment.
(3) Differences in bacterial diversity among vegetation types were insignificant, but community composition differed significantly (PERMANOVA, *p* = 0.0001). Nevertheless, these compositional differences may be confounded by within-group multivariate dispersion (PERMDISP, *p* = 0.0129). In contrast, fungal diversity demonstrated significant variation; however, the samples were relatively concentrated in the PCoA plot, indicating that vegetation type significantly affects fungal community diversity but does not correlate significantly with changes in community composition.
(4) TOC, Mn, TP, and Fe in the RDA were all closely related to the bacterial community; however, among them, Mn concentration was the environmental variable with the highest marginal explanatory power (marginal R^2^ = 19.29) and its effect on the bacterial community reached a significant level (BH-q = 0.0024). Mn concentration was positively correlated with the ACE, Chao1, and Simpson indices and negatively correlated with the Shannon index, indicating that increasing Mn concentration enhanced the richness of the bacterial community while reducing its evenness. TOC and SWC in the RDA were closely related to the fungal community; however, TOC exhibited the highest marginal explanatory power (marginal R^2^ = 13.66), although its effect on the fungal community did not reach a significant level (BH-q = 0.1048). TOC was negatively correlated with the ACE, Chao1, and Shannon indices but positively correlated with the Simpson index, indicating that high concentrations of TOC simultaneously decreased both the richness and evenness of the fungal community.

## Figures and Tables

**Table 1:** Environmental variables related to soil microorganisms.

Environmental Variables	MF	LR	MR	AF
SWC (%)	30.55 ± 6.08 a	33.61 ± 6.48 a	27.85 ± 7.22 a	30.13 ± 2.65 a
BD (g/cm^3^)	1.40 ± 0.29 a	1.41 ± 0.22 a	1.44 ± 0.24 a	1.33 ± 0.16 a
pH	5.52 ± 0.22 a	5.01 ± 0.23 c	5.36 ± 0.21 ab	5.27 ± 0.15 b
TN (g/kg)	1.34 ± 0.69 a	1.25 ± 0.53 a	1.52 ± 0.66 a	1.14 ± 0.43 a
TOC (g/kg)	17.14 ± 9.16 a	15.21 ± 5.58 a	18.62 ± 9.58 a	14.23 ± 6.76 a
TP (g/kg)	0.17 ± 0.09 a	0.10 ± 0.04 b	0.22 ± 0.08 a	0.09 ± 0.03 b
Mn (g/kg)	0.49 ± 0.13 b	0.25 ± 0.15 c	0.74 ± 0.20 a	0.06 ± 0.04 d
Fe (g/kg)	18.83 ± 2.79 b	18.28 ± 5.59 bc	25.67 ± 6.37 a	13.56 ± 2.67 c
El (m)	306.14 ± 110.11 c	500.71 ± 179.28 b	1020.86 ± 80.69 a	584.57 ± 184.94 b

Note: Different letters in the same row indicate significant differences at the 0.05 significance level. MF, LR, MR, and AF represent monsoon forests, lowland rainforests, montane rainforests, and artificial forests, respectively. SWC, BD, TN, TOC, and TP denote the soil water content, bulk density, total nitrogen, total organic carbon, and total phosphorus, respectively. Mn and Fe represent manganese and iron concentrations, respectively. El indicates elevation.

**Table 2:** Diversity Indices Under Different Vegetation Types.

	Type	ACE Index	Chao1 Index	Shannon Index	Simpson Index
Bacteria	MF	4567.58 ± 32.94 a	4593.09 ± 37.57 a	5.03 ± 0.08 a	0.05 ± 0.01 a
	LR	4599.31 ± 49.41 a	4624.65 ± 55.76 a	5.05 ± 0.14 a	0.06 ± 0.01 a
	MR	4592.50 ± 25.85 a	4626.26 ± 28.31 a	5.09 ± 0.13 a	0.05 ± 0.01 a
	AF	4565.45 ± 46.22 a	4583.37 ± 50.24 a	5.17 ± 0.12 a	0.04 ± 0.01 a
Fungi	MF	510.75 ± 64.82 b	518.88 ± 68.28 b	3.83 ± 0.61 b	0.10 ± 0.09 a
	LR	606.39 ± 33.23 a	619.75 ± 41.75 a	4.52 ± 0.16 a	0.03 ± 0.01 b
	MR	526.79 ± 35.84 b	533.34 ± 34.63 b	4.36 ± 0.18 ab	0.06 ± 0.06 ab
	AF	582.49 ± 18.53 a	585.94 ± 29.81 a	4.30 ± 0.17 ab	0.06 ± 0.04 ab

Note: Different letters in the same column indicate significant differences at the 0.05 significance level. MF, LR, MR, and AF denote monsoon forests, lowland rainforests, montane rainforests, and artificial forests, respectively. El indicates elevation.

**Table 3:** Marginal explanatory power and significance test results of environmental variables.

Microorganism	Environmental Variables	Original Marginal R^2^ (%)	F	*p*	BH-q
Bacteria	Mn	19.29	14.18	0.0003	0.0024
	Fe	4.61	3.39	0.0316	0.0828
	El	4.59	3.38	0.0414	0.0828
	TOC	4.15	3.05	0.0361	0.0828
	pH	2.76	2.03	0.1119	0.1790
	TP	1.86	1.37	0.2331	0.3108
	SWC	1.38	1.01	0.3521	0.4035
	BD	0.62	0.46	0.7127	0.7127
Fungi	TOC	13.66	5.77	0.0131	0.1048
	SWC	4.91	2.07	0.1363	0.3963
	Mn	4.56	1.92	0.1486	0.3963
	BD	2.77	1.17	0.3006	0.6012
	Fe	2.17	0.92	0.3906	0.6188
	El	1.51	0.64	0.5184	0.6188
	pH	1.23	0.52	0.5921	0.6188
	TP	1.06	0.45	0.6188	0.6188

Note: SWC, BD, TN, TOC, and TP denote the soil water content, bulk density, total nitrogen, total organic carbon, and total phosphorus, respectively. Mn and Fe represent manganese and iron concentrations, respectively. El indicates elevation.

## Data Availability

The raw data used in the paper are subject to confidentiality requirements and cannot be made publicly available. For further inquiries, please contact the corresponding author.

## Supplementary Materials

The following supporting information can be downloaded at https://www.mdpi.com/article/10.3390/biology15211968/s1, Table S1: Sample Information Table; Table S2: Relative Abundance of Soil Bacteria at the Phylum Level; Table S3: Relative Abundance of Soil fungi at the Phylum Level.

## Abbreviations

The following abbreviations are used in this manuscript:

MF	Monsoon Forests
LR	Lowland Rainforests
MR	Montane Rainforests
AF	Artificial Forests (Specifically referring to the artificial forests included in this research)
