# Supplementary material for: Characteristics of Soil Microbial Communities and Their Associated Environmental Variables Under Different Vegetation Types in Jianfengling, Hainan
Source: Biology (Basel). 2026 Nov 2;15(21):1968. doi: 10.3390/biology15211968 (PMC13762156; doi:10.3390/biology15211968)
Supplement: Supplementary file 1 [file biology-15-01968-s001.zip › biology-4543121-supplementary.pdf]

Table S1. Sample Information Table

Samples	Vegetation type	raw reads	clean reads	Raw base (bp)	Clean base (bp)	N50	Contigs	Contigs bases (bp)	mapping rate （%）	ORFs	Bacteria Good's coverage	Fungi Good's coverage
1	LR	42596758	42376718	6432110458	6378514261	732	174544	134110710	18.47	239863	0.99999	0.99194
2	LR	45323898	45102156	6843908598	6789881846	748	198700	157629178	17.42	272044	0.99999	0.98963
3	LR	41319346	41115838	6239221246	6190123751	745	157717	124845483	17.35	216434	0.99999	0.98537
4	LR	40469670	40277406	6110920170	6064616035	771	228483	183024715	24.39	312380	0.99999	0.99207
5	LR	43426630	43207192	6557421130	6505471383	726	153531	116113765	13.62	205172	0.99998	0.98608
6	LR	48893542	48639526	7382924842	7321459848	726	188566	143734902	15.72	252645	0.99999	0.98814
7	LR	43574566	43340534	6579759466	6524220522	735	202587	154831595	18.56	271590	0.99999	0.98579
8	LR	44128180	43887316	6663355180	6606356900	772	142872	118645334	14.48	204920	0.99999	0.98623
9	LR	43225906	42996200	6527111806	6472762874	702	183159	133593566	14.43	240042	0.99999	0.98778
10	LR	39506572	39306742	5965492372	5917482489	659	91337	62776862	7.77	115087	0.99998	0.98668
11	LR	46610916	46357004	7038248316	6978424377	727	165958	127462126	13.65	222247	0.99998	0.98671
12	LR	40211650	39987736	6071959150	6016401770	743	183380	142540288	20.93	244090	0.99999	0.98781
13	LR	40151090	39931578	6062814590	6012563318	756	222591	174921991	24.16	302496	0.99999	0.99161
14	LR	41174612	40949736	6217366412	6163774362	760	201715	160381364	20.01	274340	0.99999	0.99103
15	MR	44516944	44270294	6722058544	6662261771	767	177913	144151540	17.88	255621	0.99999	0.98343
16	MR	46196248	45921326	6975633448	6910907699	815	149472	128889169	15.19	219077	0.99998	0.98655
17	MR	46230198	45953488	6980759898	6915945285	784	276242	224232277	25.56	395253	0.99999	0.98708
18	MR	41779046	41558276	6308635946	6257281890	745	175340	139245040	17.10	243517	0.99998	0.99041
19	MR	45397796	45155430	6855067196	6796944519	765	220530	178838623	20.11	310245	0.99999	0.98669
20	MR	48247216	47993620	7285329616	7223855041	732	128763	101866482	11.50	178641	0.99998	0.98378
21	MR	43627238	43378830	6587712938	6528136050	818	157942	135713777	16.14	229633	0.99998	0.98233

Samples	Vegetation type	raw reads	clean reads	Raw base (bp)	Clean base (bp)	N50	Contigs	Contigs bases (bp)	mapping rate （%）	ORFs	Bacteria Good's coverage	Fungi Good's coverage
22	MF	40582094	40359108	6127896194	6072986330	901	146823	137842370	19.74	228349	0.99998	0.98749
23	MF	45073418	44838018	6806086118	6748584351	746	218449	170591731	20.82	302185	0.99999	0.98865
24	MF	42838940	42589182	6468679940	6409931110	760	142083	117136789	14.37	203154	0.99998	0.98038
25	MF	43745742	43500678	6605607042	6545492052	727	208274	157781951	20.49	284528	0.99999	0.99837
26	MF	43025950	42786252	6496918450	6439623433	797	189976	158751357	20.97	274173	0.99999	0.98588
27	MF	42112302	41891174	6358957602	6306773460	728	130218	100338146	13.29	176844	0.99999	0.98335
28	MF	44265504	44023618	6684091104	6625348973	698	145484	106461471	13.04	193133	0.99998	0.98591
29	AF	43476586	43258266	6564964486	6513751192	824	299282	253265888	31.64	421930	0.99999	0.99143
30	AF	42819514	42619432	6465746614	6417913271	826	316520	266279972	30.25	447139	0.99999	0.99455
31	AF	45018650	44802896	6797816150	6748981401	779	266351	214474086	25.88	367560	0.99999	0.99255
32	AF	47944864	47722926	7239674464	7185122757	795	318212	259980303	28.60	441921	0.99999	0.99419
33	AF	47078140	46851738	7108799140	7054852677	785	317233	258633019	32.79	441880	0.99999	0.9945
34	AF	43646012	43415014	6590547812	6536593651	778	289310	233662925	31.63	400319	0.99999	0.99398
35	AF	46461812	46222664	7015733612	6957472009	788	298576	243031417	30.89	420084	0.99999	0.99342

Note: MF, LR, MR, and AF represent monsoon forests, lowland rainforests, montane rainforests, and artificial forests, respectively.

Table S2. Relative Abundance of Soil Bacteria at the Phylum Level

Phylum	MF (%)	LR (%)	MR (%)	AF (%)
Pseudomonadota	44.15	48.53	42.90	54.20
Actinomycetota	29.86	21.36	29.03	10.61
Acidobacteriota	3.58	8.56	4.37	15.23
Verrucomicrobiota	7.38	6.68	7.50	5.65
Vulcanimicrobiota	1.43	3.36	1.78	5.25
Chloroflexota	1.79	2.00	2.13	0.84
Candidatus_Rokuibacteriota	2.69	1.38	2.68	0.68
Planctomycetota	0.95	1.17	0.91	1.37
Myxococcota	0.98	1.05	1.06	0.96
Bacillota	1.11	0.89	1.32	0.68
Cyanobacteriota	0.78	0.80	0.76	0.98
Candidatus_Dormiibacterota	0.60	0.62	1.02	0.12
Others	4.70	3.60	4.54	3.43
Total	100	100	100	100

Note: MF, LR, MR, and AF represent monsoon forests, lowland rainforests, montane rainforests, and artificial forests, respectively.

Table S3. Relative Abundance of Soil fungi at the Phylum Level

Phylum	MF (%)	LR (%)	MR (%)	AF (%)
Ascomycota	52.25	59.74	70.41	65.08
Basidiomycota	33.66	17.62	9.86	19.35
Mucoromycota	11.35	18.85	16.25	11.18
Chytridiomycota	1.96	2.54	2.6	3.29
Zoopagomycota	0.4	0.65	0.67	0.815
Microsporidia	0.31	0.45	0.07	0.2
Blastocladiomycota	0.04	0.12	0.09	0.08
Cryptomycota	0.02	0.02	0.03	0.003
Olpidiomycota	0.01	0.01	0.02	0.002
Total	100	100	100	100

Note: MF, LR, MR, and AF represent monsoon forests, lowland rainforests, montane rainforests, and artificial forests, respectively.
